# Is Early Childhood Development Care at Public Health Facilities in Pakistan Effective? A Cluster Randomized Controlled Trial

**DOI:** 10.9745/GHSP-D-23-00037

**Published:** 2023-10-30

**Authors:** Nida Khan, Muhammad Amir Khan, Muhammad Ahmar Khan, Amna Ejaz, Azza Warraitch, Sehrish Ishaq, Ehsan Salahuddin, Haroon Jehangir Khan, John D. Walley

**Affiliations:** a Association for Social Development, Islamabad, Pakistan.; b Directorate General of Health Services, Punjab, Pakistan.; c Nuffield Centre for International Health and Development, Leeds Institute of Health Sciences, University of Leeds, United Kingdom.

## Abstract

**Background:**

Significant brain development in children occurs from birth to 2 years, with environment playing an important role. Stimulation interventions are widely known to be effective in enhancing early childhood development (ECD). This study aims to assess the feasibility and effectiveness of integrating ECD care delivered by lady health visitors (LHVs) at public health facilities in rural Pakistan.

**Method:**

A cluster randomized controlled trial was conducted through public health facilities in 2 districts of Punjab, Pakistan. A total of 22 clusters (rural health centers and subdistrict hospitals) were randomly allocated to receive routine care (control: n=11 clusters, 406 mother-child pairs) or counseling (intervention: n=11 clusters, 398 mother-child pairs). All children aged 11–12 months without any congenital abnormality were eligible for enrollment. The intervention was delivered by the LHVs to mothers with children aged 12–24 months in 3 quarterly sessions.

**Results:**

The primary outcome was the prevention of ECD delays in children aged 24 months (assessed with the Ages and Stages Questionnaire-3). Analysis was done on an intention-to-treat basis. A total of 804 mother-child pairs were registered in the study, of which 26 (3.3%) pairs were lost to follow-up at the endpoint. The proportion of children with 2 or more developmental delays was significantly less in the intervention arm (13%) as compared to the control arm (41%) at an endpoint (odds ratio=0.21; 95% confidence interval=0.11, 0.42). Children in the intervention arm also had significantly better anthropometric measurements when aged 24 months than the children in the control arm.

**Conclusion:**

The integrated ECD care intervention for children aged 12–24 months at public health facilities was found to be effective in enhancing ECD and reducing the proportion of children with global development delays.

## Abstract (precis)

An integrated early childhood development care intervention effectively reduced global development delays and improved growth outcomes in rural Pakistan.

## Teaser

**Key Findings:**

- The integrated early childhood development (ECD) care package for public health facilities was found to be effective, leading to a reduction in global development delays and improvement in growth outcomes within the low-resource setting in Pakistan.
- The pragmatic study design shows that lady health visitors can successfully integrate ECD into their routine work.

**Key Implications:**

- The integration of ECD care at public health care facilities can successfully reduce global development delays among children aged 12–24 months.
- The study indicates promising evidence for the scale-up and further integration of ECD interventions in the existing primary and secondary care in similar low- and middle-income country contexts.

## BACKGROUND

A 2017 Lancet series on early childhood development estimated that around 250 million children in low- and middle-income countries (LMICs) are at risk of developmental delays^1^ due to multiple biological and psychosocial risk factors prevalent in LMICs.^2^ Recent studies in LMICs indicate a prevalence of developmental delays ranging from 25%^3^ to 43%.^4^ In Pakistan, approximately 8 million children have unmet development potential, 40% of children are stunted, every 1 of 3 children are underweight, and 18% of children are wasted.^5^ Statistics indicate that 30% of children in families with low socioeconomic status have developmental delays.^6^

Some of the most common risk factors of child developmental delays are associated with poverty.^2^^,^^7^ This leads to social inequalities as children at risk of developmental delays experience difficulties in academic achievement that limit future economic earning opportunities, thus trapping them in a cycle of poverty, which affects the social and economic development of society.^8^^,^^9^ Acknowledging the severe consequences of early child developmental delays, early childhood development (ECD) is now considered a global health priority as demonstrated by Sustainable Development Goal 4.2, which states that all children should have access to “quality early childhood development, care and pre-primary education.”^10^

Brain development occurs more rapidly during the first 2 years of life through the interaction of multiple biological and psychosocial stimuli.^11^ Burgeoning evidence has established the effectiveness of different psychosocial interventions to address the modifiable risk factors for developmental delays.^12^ The active ingredients of these interventions include providing optimal nutrition to the child, providing age-appropriate stimulation using play-based exercises, and improving mother-child interaction by training mothers to be more responsive to their child's signals.^13^^,^^14^

In recent years, multiple studies have been conducted to explore the efficacy of different ECD programs in LMICs, including Pakistan.^15^^,^^16^ The gap in the unmet potential for child development globally has recently been attributed to the failure to scale up the ECD interventions using a multisector approach.^1^ The 2017 Lancet series^1^ indicated a need to integrate health care programs into the existing public health and nutrition services to maximize the reach and effectiveness of ECD programs because the public health care system is the first source of contact for most of the population in LMICs.^17^ However, little evidence exists on the effectiveness of integrating ECD interventions into programs. A rigorous evaluation of integrated ECD care packages in the public health care system is needed^18^ to assist the policymakers to scale up integrated ECD care at the national level.

The gap in the unmet potential for child development globally has recently been attributed to the failure to scale up the ECD interventions using a multisector approach.

The Non-Communicable Diseases-Maternal Health Strategic Plan of the Punjab Department of Health has recognized ECD integration into the maternal and child health (MCH) services currently provided at primary public health care facilities as a provincial priority.^19^ To address this service and knowledge gap, we developed a contextualized ECD care package for integration into the ongoing MCH services at public health care facilities in Pakistan. The public health facilities in Punjab province comprise teaching hospitals, district hospitals, tertiary hospitals, rural health centers (RHCs), and basic health units. Every public health facility has at least 1 appointed lady health visitor (LHV), who is responsible for basic nursing care; maternal, newborn, and child health; and the training of community workers. We integrated the ECD care package in all eligible RHCs and tertiary hospitals in 2 districts of Punjab. The package included a training manual for LHVs, a pictorial flipbook for counseling mothers, and take-home brochures.

This study aimed to evaluate the effectiveness of the integrated ECD care package in reducing the prevalence of global developmental delays among children aged 24 months old as compared to the routine care provided at public health care facilities.

## METHODS

### Study Design

A pragmatic, parallel, cluster randomized controlled trial (cRCT) was conducted to evaluate the effectiveness of an integrated ECD care package at public health facilities in Punjab, Pakistan. The unit of randomization was a public health facility at a 1:1 allocation ratio. Cluster design was used to prevent the risk of contamination as it was not viable to expect the provider to effectively give or withhold the components of the intervention based on the client's individual allocation. The reporting adhered to the CONSORT extension for randomized trials (The CONSORT 2010 Checklist).^20^

### Study Settings

**FIGURE 1:**
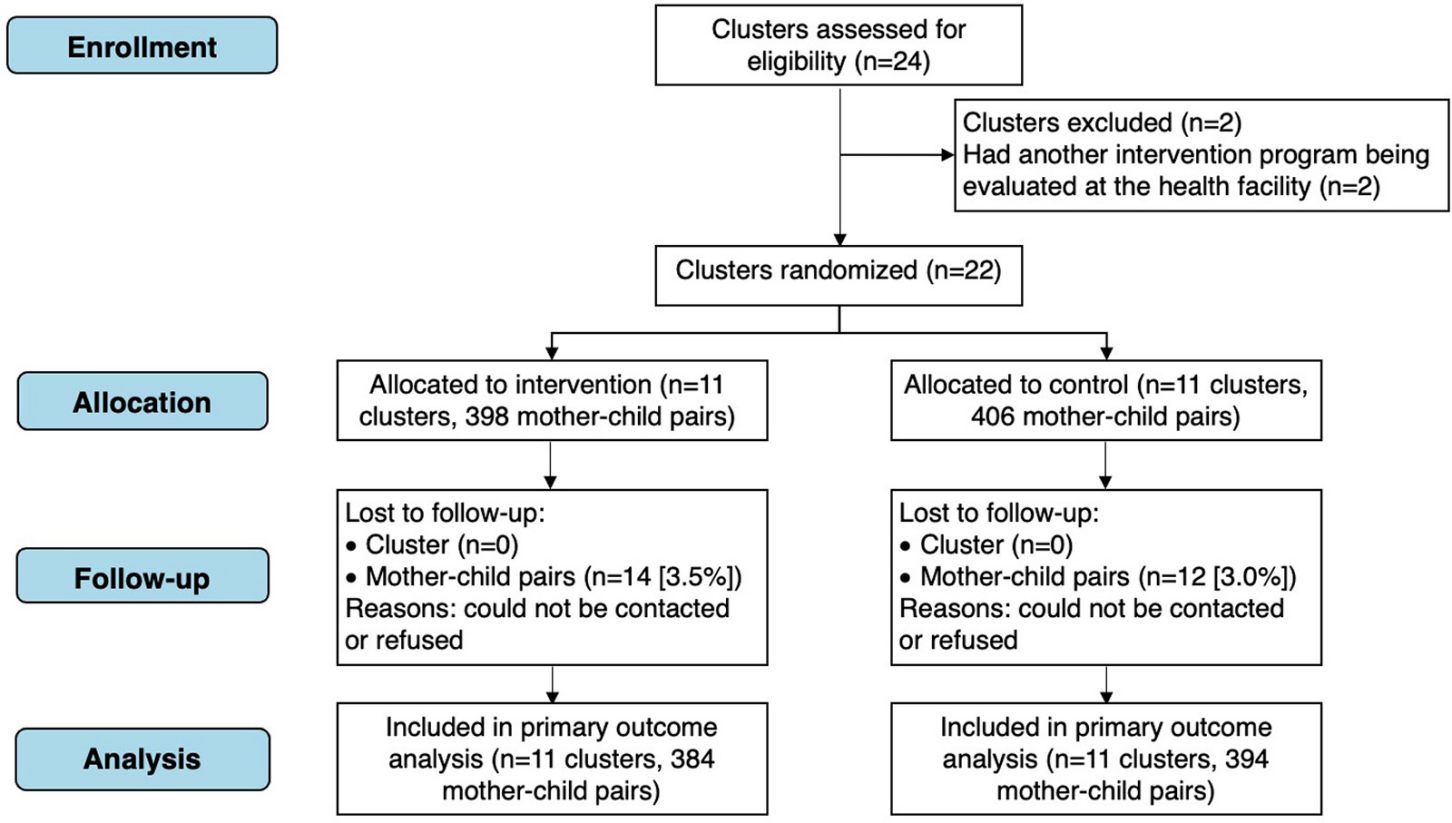
CONSORT Flow Diagram of Cluster Selection to Evaluate Effectiveness of the Integrated Early Childhood Development Care Intervention in Punjab, Pakistan

This cRCT was conducted from December 2017 to January 2019 in 2 districts of Punjab (Rawalpindi and Lahore). These 2 districts were selected in consultation with the Directorate General of Health Services. The estimated population of these 2 districts is approximately 16 million.^21^ The 2 selected districts include 24 functioning public health care facilities (i.e., 16 RHCs and 8 secondary hospitals). These public facilities were eligible for participation in this study if (1) the health facility in-charge gave consent to the participation of the site and staff in the study, (2) the facility was providing routine MCH services, and (3) it did not have any alternate mental health care intervention program under evaluation during the time of the study. All 24 facilities were assessed for eligibility, and 2 were excluded because of an ongoing World Health Organization mental health action gap program pilot study being conducted at those facilities. Twenty-two remaining facilities were offered and included in the study (Figure 1). At each cluster, an average of 10 mothers with children aged 12 months (± 29 days) visiting the outpatient department resulted in an estimated average cluster size of 30 children during the recruitment period of 3 months. A total of 804 mother-child dyads from 22 clusters were recruited in the study.

### Study Participants

Mother-child dyads were eligible to participate if the child was aged 11–12 months (i.e., 11 months and 1 day to 12 months and 29 days) and the mother provided written informed consent. Children with a history of congenital abnormality or mothers intending to move out of the area during the study period were excluded from the trial. Mother-child dyads visiting these health facilities for any reason were enrolled sequentially, and recruitment was stopped when the required number of registrations in the respective trial arm was achieved. All enrolled mother-child dyads were followed up until 1 year.

### Study Procedures

Mother-child dyads were assessed for eligibility using an eligibility criteria checklist, and those meeting the criteria were oriented on the study purpose and procedures by the LHVs using an informed consent form in the national language (Urdu). Those who gave written informed consent were included in the study, marking their first visit as the baseline visit. LHVs measured and recorded children's age (days), height (inches), and weight (kg) at baseline visits. The measurement of children's weight and height was done using standardized protocols and recorded on growth monitoring charts. The children with severe physical conditions were referred.

### Intervention Design

The intervention was developed in consultation with a technical working group composed of public health experts, pediatricians, nutritionists, child psychiatrists, developmental disorder experts, LHVs, and mothers.

In the intervention arm, at least 1 LHV at each facility was given a 2-day training on delivering the intervention package. The competence of the LHV to deliver the ECD care was assessed using an adapted checklist. LHVs scoring 80% or above on the competence checklist were allowed to deliver ECD care. LHVs scoring less than 80% were retrained until they met the threshold for competence. The fidelity of LHV was assessed through observation by project staff.

The LHVs delivered the ECD care package to each mother-child dyad individually in 3 quarterly sessions. The average duration of each session was 30 minutes. Sessions were conducted in developmental order, starting from the chronological age level and progressing to the next session on a quarterly basis. Each session comprised age-appropriate key strategies to enhance children's mental health and physical growth (Table 1). Mothers were counseled to implement these strategies with their children. The counseling was given using a pictorial flipbook, and mothers were given take-home brochures. During the session, the LHVs also demonstrated to mothers the ideal implementation of strategies and exercises to be conducted with children. The strategies were adapted to ensure feasible implementation in the daily routine using daily life objects and activities. The mothers attended 3 follow-up sessions at the RHC on a quarterly basis. The intervention sessions were delivered on an individual basis by LHVs. The average duration of each session was 30 minutes. The counseling was given using a pictorial flipbook, and mothers were given take-home brochures.

**TABLE 1.** Key Messages of the Integrated Early Childhood Development Care Intervention in Punjab, Pakistan

Session and Child Age	Dimension	Key Messages
Session 1: 12 months	Mental health	Engage your child in a playful activity (hide-and-seek) and guide him/her to find you through your voice. Appreciate your child for participating and helping with routine activities. Help your child identify new objects and words with the help of images. Encourage your child to get involved in everyday chores by giving him/her easy instructions. Show your child a colored shoe or sock and ask him/her to find the other.
	Physical health	Encourage your child to balance on 1 leg while you hold his/her hands. Take your child out for a walk while holding his/her finger (as per the child's needs). Encourage him/her to follow simple instructions and assist them in identifying new items as they walk. Urge the child to squeeze a damp sponge or piece of cloth. Encourage the child to collect their toys in a basket and turn it over. Encourage your child to imitate your bodily movements and show him/her your appreciation for it (by clapping hands, rolling out your tongue).
	Nutrition	Make your child eat his/her meals 3 times a day with the family. As per the child's need, soften the food bits. Breastfeed the child if he/she is hungry in between the 2 meals. A healthy diet keeps the child disease-free. To keep the food clean, wash your hands with soap, use clean utensils, and cover food before having/preparing it.
Session 2: 16 months	Mental health	Encourage your child to eat his/her meals on their own while having food with all the family members. Encourage your child to push his/her arms through sleeves and legs through pants while changing clothes. Help your child to learn and pronounce new words by showing him/her items and describing them. Show your child animals and imitate their noises to help him/her recognize new sounds. Also, encourage your child to imitate these sounds, such as "cat meows." Show your child an object, and ask him to identify similar objects in the house such as a fan, chair, etc.
	Physical health	Show the child how to kick a ball and encourage him/her to kick and then chase it themself. Instruct the child to pick an object, bring it, and then put it back. Play with blocks with your child and while counting teach him/her how to put 1 block over the other. Encourage your child to doodle on a piece of paper. Show your child how to arrange 2 or more items in an order, then have them repeat it.
Session 3: 20 months	Mental health	Show your child how to wash her/her hands with soap by opening the tap and encouraging them to repeat it. Encourage your child to engage in daily household chores while playing with toys. Encourage your child to narrate to his/her family about a significant incident from the day. Encourage your child to use a 2- or more word sentence to describe what they need, such as "give water/ I need water." Help them to recognize new words and concepts such as empty, full, straight, and tilted while playing with cups and sand.
	Physical health	Encourage your child to catch you while running in the park/garden. Encourage your child to jump from a small step/stair. Encourage your child to mix 2 or more food items in the bowl. Encourage your child to build a tower out of blocks by stacking them on top of 1 another. Draw 2 horizontal lines on a piece of paper and encourage your child to draw vertical lines in between them.

The intervention content was based on the Ages and Stages Questionnaire-3 (ASQ-3) learning activities manual.^22^ These learning activities were reviewed and adapted to suit the cultural context of Pakistan by the technical working group. The implementation modalities were also finalized with the working group, considering the local context. The piloting was conducted at 2 RHCs with 10 mother-child dyads. The pilot informed the intervention design and content. Piloting the amount of intervention content indicated that delivery of 2 key messages per 5 domains of child development was optimal. Mothers reported difficulties in retaining and implementing content at levels higher than that amount. The physical growth was assessed using anthropometric measures. The components in physical growth also included assessment and response to (1) protein-energy malnutrition and (2) the child's micronutrient status, which included iron deficiency, vitamin A deficiency, vitamin D deficiency, and iodine deficiency. The treatment for these deficiencies primarily consisted of oral supplements.

In the control arm, LHVs provided routine care. To ensure LHVs took standardized measurements as well as recording and reporting, they received a 1-day training on taking and recording children's anthropometric measurements using growth monitoring cards. However, the training was limited to measurements, recording, and reporting and did not include any standardized intervention care on child development. The LHVs took and recorded the anthropometric measurements at every follow-up visit in the control arm.

### Outcomes

#### Primary Outcome

Our primary outcome was a reduced prevalence of global developmental delays in the intervention arm as compared to the control arm when the child was aged 24 months, measured using the ASQ-3.^23^ A global developmental delay is defined as significant delay in 2 or more child developmental domains (i.e., communication, gross motor, fine motor, problem-solving and personal-social).^24^ Continuous scores for the 5 domains can be calculated by calculating a sum of individual items; however, calculation of an overall score for the total ASQ scale by combining scores of 5 domains is not recommended.

This questionnaire enables the identification of development delays accurately at an early stage and so can be referred for early-level interventions. It has strong psychometric properties with 96% specificity, 87% sensitivity, and 92% test-retest reliability and has been examined for screening development delays in LMICs.^25^ The tool has been translated into Urdu and used in Pakistan previously.^16^ The cutoffs for each domain were used as given in the ASQ-3 manual (i.e., the score of 25.17 for communication, 38.07 for gross motor, 35.16 for fine motor, 29.78 for problem-solving, and 31.54 for personal/social).^23^ Any score below these cutoffs was recorded as a developmental delay. ASQ-3 was only administered at the endpoint as it was deemed unfeasible for the LHVs to administer the ASQ at baseline under programmatic conditions. The client was defined as adherent if they had attended all 3 follow-up visits and taken the counseling sessions.

#### Secondary Outcome

The secondary outcomes assessed were height-for-age z-scores, weight-for-age z-scores, weight-for-height z-scores, and stunting, which was defined as height-for-age z-scores<−2 as calculated using the 2006 World Health Organization child growth standards.^26^

At the baseline and quarterly follow-up visits in both arms, children's length and weight were measured by the trained LHVs as per standardized protocols of World Health Organization child growth standards.^26^ Children's length was measured lying down using a length board or infantometer to the nearest 0.1 cm. At baseline and quarterly follow-up visits, weight was measured using the LAICA baby scale. The anthropometric measurements were recorded on the growth charts provided at health facilities.

Endpoint assessments were conducted by trained external evaluators holding an undergraduate degree in psychology. All external evaluators were extensively trained on the standardized protocols for measuring a child's development using the ASQ-3 and physical growth (i.e., height and weight). Inter-rater reliability of external evaluators was examined before conducting the endpoint assessment. External evaluators started the endpoint assessments after meeting the threshold of acceptable inter-rater reliability at intracluster correlation>0.80. All endpoint assessments were conducted at the public health facilities in a dedicated room. LHVs requested the mothers via a call to visit the health facility at a specified date (+7 days after a child turned 24 months). Upon visiting the health facility, the mothers were oriented about the endpoint procedure and instructed using standard narrative to not disclose any information about the type of treatment received to the endpoint evaluators.

During the endpoint assessment, height was measured using a height board or stadiometer mounted at a right angle between the leveled floor and against a wall. Children were weighed alone using the LAICA baby scale, and growth charts were used to record anthropometric measurements. The ASQ-3 was administered using the interview method due to the low literacy rate of mothers. To ensure standardization of question wording, if a participant had trouble understanding a question, the question was repeated for the mother. Caregiver-rated ASQ-3 was administered instead of observation based to reduce the risk of observation bias. As per our previous experience, children are often underrated in observations because they tend to underperform during the limited time frame specified for observation by an independent observer. The novelty of observation scenarios and unfamiliarity with the independent observer further hinders the observation process. Because caregivers spend the majority of their time with children and are knowledgeable about children's functioning and developmental milestones, it was perceived as the most productive mode of evaluation for children's development.^27^ The mothers who could not be contacted at the follow-ups or endpoint for more than 1 month were considered as lost to follow-up.

### Sample Size

The sample size was calculated using the cluster-level power calculations command in Stata version 14.2. A sample of 330 children per arm was estimated at 80% power and 0.05 significance level to detect the change in prevalence of global developmental delays from 32% in the control arm to 17% in the intervention arm at the endpoint,^16^ using 0.04 intracluster correlation^28^ and an average cluster size of 30 mother-child dyads from 22 clusters. The attrition rate at the endpoint was assumed to be 20%, resulting in a total required sample of 792 mother-child dyads.

### Randomization and Masking

**FIGURE 2:**
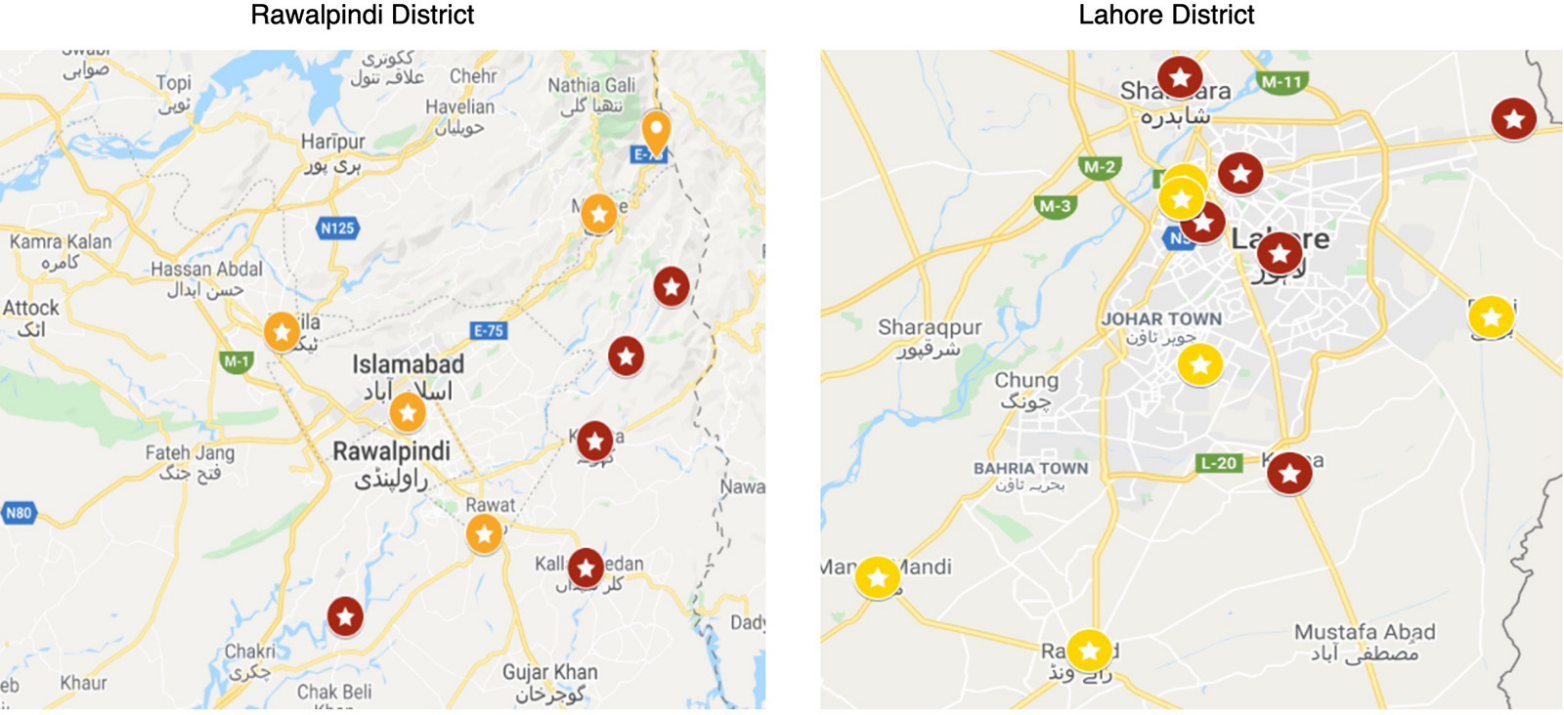
Map of Health Care Facilities^a^ in Rawalpindi District and Lahore District, Pakistan, Participating in the Integrated Early Childhood Development Care Intervention ^a^ Red stars indicate facilities in the control group; yellow stars indicate facilities in the intervention group.

To minimize the risk of contamination between research participants, we defined the unit of randomization as a public health facility. Each cluster was assessed for eligibility by the research team. Twenty-two of 24 eligible clusters were randomized into intervention and control arms on a 1:1 allocation ratio before the recruitment of study participants from each cluster. Upon finalizing the list of eligible clusters, the project manager allocated a trial ID that was then randomized to the intervention or control arm. The randomization was conducted by an independent statistician using Stata, generating a randomized code list. Figure 2 shows the geographical locations of the health facilities for each group.

The external evaluation team was blind to the allocation status of the clusters. To reduce familiarity and minimize the risk of unblinding, schedules were updated to change the public health facility assigned to each assessor every week during the endpoint assessments. The independent statistician analyzing the outcome data was also blind to the allocation status of clusters.

### Statistical Analysis

Statistical analyses were conducted using SPSS version 26 and Stata version 14.2. To minimize data errors, quality assurance procedures were implemented, which included training data entry operators and checking data entry quality at regular intervals. Descriptive statistics were conducted regularly to ensure data consistency and integrity. Data privacy was ensured by removing identifiable information of participants and saving the data in password-protected computers and locked cupboards. Only the data management team had access to the study data. Robust methods suitable for cluster trials with relatively few clusters per arm^29^ were used to analyze the data. Analyses were conducted on both cluster-level summaries and individual data adjusted for clustering effects. The analysis was done on an intention-to-treat basis. A complete case analysis was done, excluding all mother-child pairs from the analysis for which outcome or covariate data was missing, depending on the data required in the analysis. A statistical significance level of 5% was set, and 2-sided *P* values were calculated. There were no interim analyses planned or conducted.

#### Analysis of Cluster-Level Summaries

The binary outcomes underwent a crude analysis that calculated cluster-level proportions followed by an independent t-test to estimate the treatment effect as the risk difference (i.e., the absolute difference in outcome proportions) between the 2 arms at the endpoint, with associated 95% confidence interval (CI) and *P* value. The continuous outcomes were analyzed in a crude analysis by calculating cluster-level means followed by an independent t-test to estimate the treatment effect as the mean difference in the cluster-level outcome values between the 2 arms (control-intervention) at the endpoint, with associated 95% CI and *P* value.

#### Analysis of Individual Data Adjusting for the Clustering Effect

We estimated logistic regression models for binary outcomes using generalized estimating equations with an exchangeable correlation structure to adjust for clustering effects. We present an unadjusted analysis comparing the intervention and control group at the endpoint, controlling only for clustering. Covariates were not added to the model, as the intervention and control arm did not differ on any baseline demographic characteristic. The standard errors of parameters in the generalized estimating equations were estimated using the bootstrap method (n=1,000) to account for the small number of clusters (n<50).^30^ Odds ratios (95% CIs) for developmental delays were calculated from variable estimates.

Linear mixed models were estimated for continuous outcomes using generalized estimating equations with an exchangeable correlation structure to adjust for clustering effects and the bootstrap method (n=1,000) for standard errors to account for the small number of clusters. The beta coefficient is the standardized coefficient showing the effect size of treatment on the outcomes.

### Ethical Approval

Ethical approval was obtained by the Pakistan National Bioethics Committee (NBC255), and the cRCT was registered in the ISRCTN registry as ISRCTN14396904. The trial protocol was published in 2021.^31^

## RESULTS

From November 10, 2017 to March 7, 2018, 804 children aged 12 months (±15 days) were recruited in 22 public health facilities. The last follow-up was completed on February 26, 2019. There was a similar loss to follow-up in both arms: 14 (3.5%) mother-child pairs in the intervention arm and 12 (3%) mother-child pairs in the control arm. Table 2 shows the baseline demographic characteristics of the clusters and study participants. The baseline characteristics of clusters and participants in both arms were similar and did not differ significantly between groups. Most mothers were aged in their late 20s with a mean education of 5 years, which marks the primary level of education in Pakistan. The majority of mothers were housewives. The enrolled child was, on average, the only child in the family aged younger than 5 years. More than half of the mother-child dyads lived in extended families. The mean monthly family income indicated that the socioeconomic status of the average participant was low. The proportion of boys was slightly higher in the intervention arm as compared to the control arm, but the difference was not statistically significant. Children's weight-for-age and height mean z-scores at baseline were less than 1 standard deviation below the mean, while the height-for-age and weight mean z-scores were less than 2 standard deviations below the mean.

**TABLE 2.** Baseline Characteristics of Clusters and Participants in the Integrated Early Childhood Development Care Intervention, Punjab, Pakistan

Characteristics	Intervention	Control
Clusters		
Total, No.	11	11
Average cluster size (SD)	36.2 (6.8)	36.9 (6.2)
Participants		
Mother-child pairs, No. (%)	398 (49.5)	406 (50.5)
Mothers		
Age, mean (SD), years	28.1 (4.9)	28.0 (4.7)
Year of schooling, mean (SD)	5.0 (4.6)	5.8 (4.6)
Occupation (housewife) (%)	386 (97.0)	392 (96.6)
Children		
Age, mean (SD), months	12.25 (0.403)	12.39 (0.331)
Boys, No. (%)	213 (53.5)	205 (50.5)
Height-for-age z-score, mean (SD)	−1.11 (1.44)	−1.06 (1.42)
Weight-for-age z-score, mean (SD)	−0.98 (1.33)	−0.93 (1.41)
Weight-for-height z-score, mean (SD)	−0.57 (1.23)	−0.53 (1.32)
No. of children <5 years, mean (SD)	0.6 (0.6)	0.5 (0.6)
Family structure (extended vs. nuclear), No. (%)	228 (57.3)	231 (56.9)
Average monthly family income (SD), PKR	15,616 (9,859)	16,153 (10,423)

Abbreviations: PKR, Pakistan rupee; SD, standard deviation.

The primary outcome analysis shows that the intervention arm had a significantly lower proportion of global (2 or more) development delays in children aged 24 months compared to the control arm (difference: 28 percentage points; *P*=.003). The odds of children developing global developmental delay were significantly lower in the intervention arm at age 24 months (odds ratio=0.21; 95% CI=0.10, 0.42). The intracluster correlation coefficient for the primary outcome was 0.23.

The primary outcome analysis shows that the intervention arm had a significantly lower proportion of global development delays in children aged 24 months compared to the control arm.

The proportions of children with developmental delay in each of the 5 domains of child development measured by ASQ were significantly less in the intervention arm as compared to the control arm. The odds of children developing a developmental delay in any 1 of the 5 child development domains were also significantly lower in the intervention arm.

Mean scores on 5 domains of child development were measured by ASQ and were individually above the cutoff scores for developmental delays in both arms at the endpoint. The mean scores were higher in the intervention arm, and the mean difference in scores across arms was significant. Intervention effects on the continuous outcomes of ASQ were significant for all domains of child development, indicating the effectiveness of the ECD intervention in improving child development. Monitoring of follow-up sessions indicated around 84% (95% CI=0.82, 0.86) of mothers attended all follow-up sessions, while only 62% (95% CI=0.55, 0.69) of mothers in the control arm attended all follow-up sessions (Table 3).

**TABLE 3.** Primary Outcome Analyses of the Integrated Early Childhood Development Care Intervention in Punjab, Pakistan

Binary Outcomes	Intervention Clusters, Prevalence Ratio, % (95% CI)^a^ (n=11)	Control Clusters, Prevalence Ratio, % (95% CI)^a^ (n=11)	Crude Intervention-Control Difference, PP (95% CI)	*P* Value for Crude Intervention-Control Difference	Odds Ratio^b^	Bootstrap SE	*P*>|z|	Normal-Based, 95% CI
2 or more delays	13 (0.8, 0.18)	41 (0.25, 0.57)	−28 (−0.45, −0.11)	.003	0.21	0.75	.00	0.11, 0.42
Communication	8 (0.05, 0.11)	29 (0.19, 0.39)	−21 (−0.32, −0.10)	.001	0.21	0.06	.00	0.12, 0.36
Gross motor	10 (0.06, 0.15)	26 (0.12, 0.40)	−16 (−0.30, −0.02)	.031	0.32	0.13	.00	0.15, 0.71
Fine motor	11 (0.07, 0.16)	32 (0.17, 0.48)	−21 (−0.37, −0.05)	.014	0.27	0.10	.00	0.12, 0.57
Problem-solving	8 (0.02, 0.14)	28 (0.13, 0.43)	−20 (−0.35, −0.04)	.018	0.23	0.12	.00	0.08, 0.63
Personal/social	17 (0.08, 0.25)	35 (0.23, 0.48)	−19 (−0.33, −0.04)	.013	0.37	0.13	.00	0.17, 0.77

Continuous Outcomes	Mean (SD)		Crude Intervention-Control Difference, PP (95% CI)	*P* Value for Crude Intervention-Control Difference	β	Bootstrap SE	*P*>|z|	95% CI
Communication	46.63 (11.798)	40.36 (15.141)	6.27 (4.37, 8.18)		6.67	2.86	.02	1.05, 12.28
Gross motor	49.30 (10.695)	43.52 (11.115)	5.78 (4.25, 7.32)		5.89	2.31	.01	1.35, 10.44
Fine motor	44.75 (8.975)	39.91 (11.077)	4.84 (3.42, 6.26)		4.94	2.06	.01	0.90, 8.98
Problem-solving	46.77 (11.420)	38.11 (12.96)	8.66 (6.94, 10.38)		8.65	2.52	.00	3.70, 13.60
Personal/social	46.42 (11.294)	40.08 (13.549)	6.34 (4.59, 8.10)		6.45	2.55	.01	1.44, 11.47
Adherence to follow-up visits, % (95% CI)	84% (0.82, 0.86)	62% (0.55, 0.69)	22 (0.15, 0.29)	.001				

Abbreviations: CI, confidence interval; PP, percentage point; SD, standard deviation; SE, standard error.

a Arm-specific proportions and their 95% CIs are based on cluster-level summary outcomes.

b Odds ratio are calculated using generalized estimating equations on 778 observations.

The anthropometric measurements of children at the endpoint also indicated better physical child growth (i.e., z-scores for height-for-age, weight-for-age, and weight-for-height were significantly better in the intervention arm as compared to the control arm) (Table 4). Results of the linear mixed model indicated a slight effect of ECD intervention on children's growth, and this effect was significant. The proportion of children who were stunted, underweight, and wasted was also found to be lower in the intervention arm as compared to the control arm; however, this difference was not statistically significant. Odds ratios indicated children in the intervention arm had slightly less likelihood of being underweight or wasted, although this was not statistically significant.

**TABLE 4.** Secondary Outcome Analyses of the Integrated Early Childhood Development Care Intervention in Punjab, Pakistan

Continuous Outcomes	Intervention, Mean Z-Score (95% CI)^a^ (n=11)	Control, Mean Z-Score (95% CI)^a^ (n=11)	Mean Difference (95% CI)	*P* Value for Mean Difference	β	Bootstrap SE	*P*>|z|	95% CI
Height-for-age z-scores	−0.96 (−1.19, −0.73)	−1.22 (−1.32, −1.11)	0.26 (0.01, 0.50)	.042	0.29	0.10	.00	0.09, 0.48
Weight-for-age z-scores	−0.92 (−1.04, −0.81)	−1.23 (−1.42, −1.04)	0.31 (0.10, 0.52)	.007	0.32	0.09	.00	0.14, 0.51
Weight-for-height z-scores	−0.62 (−0.69, −0.54)	−0.85 (−1.06, −0.64)	0.23 (0.01, 0.45)	.040	0.22	0.10	.03	0.02, 0.43

Binary Outcomes	Proportions (95% CI)		Difference (95% CI)	*P* Value for Difference	Odds Ratio^b^	Bootstrap SE	*P*>|z|	95% CI
Moderate to severe stunting	0.33 (0.24, 0.42)	0.32 (0.24, 0.40)	0.014 (−0.10, 0.13)	.805	1.02	0.25	.91	0.63, 1.69
Moderate to severe underweight	0.31 (0.22, 0.39)	0.32 (0.22, 0.42)	−0.01 (−0.14, 0.11)	.858	0.92	0.25	.77	0.53, 1.58
Moderate to severe wasting	0.10 (0.06, 0.15)	0.16 (0.06, 0.26)	−0.05 (−0.16, 0.05)	.298	0.63	0.27	.28	0.27, 1.47

Abbreviations: CI, confidence interval; SE, standard error.

a Arm-specific proportions and their 95% CIs are based on cluster-level summary outcomes.

b Odds ratio are calculated using generalized estimating equations on 778 observations.

The effect modification analysis by sex on the primary outcome (i.e., the proportion of children with 2 or more delays) showed an insignificant difference in results among both sexes, thus implying that the primary outcome does not differ significantly by the sex of the child.

## DISCUSSION

This study shows that it is feasible to integrate an ECD care package at RHCs and subdistrict hospitals, as reflected by the enrollment rate and minimal attrition rate. Integration of similar interventions in primary care has also been found feasible and effective in many other settings, such as Jamaica, where parenting skills and early psychosocial interventions integrated at the primary care level resulted in improvement in child development and knowledge and practices of mothers.^32^ Moreover, integrating maternal and newborn care interventions into primary health care settings can contribute significantly to scalability and sustainability of such interventions.^33^

The behavioral stimulation intervention resulted in a lower proportion of children aged 12–24 months with developmental delays as compared to routine care. Similarly, various stimulation interventions in rural Pakistan, Bangladesh, and Uganda were also found to be effective in improving cognitive, language, and motor development.^15^^,^^17^^,^^29^^,^^34^ We also report the combined effect of behavioral stimulation and nutrition components. Integrating such interventions has been found to not only have additive effects on child development but also be economical and resource efficient.^15^

Our study found that mother education to promote home-prepared recipes and hygienic food preparation and storage led to significant improvement in child growth. However, this did not significantly decrease the proportion of stunted, underweight, or wasted children. A similar intervention in rural China on basic education of nutrition delivered through local health services led to better z-scores among children.^35^ Various other studies have shown strong evidence that improved care practices through parenting education are essential for optimal growth as well as psychological development.^12^^,^^36^

The strengths of our study include a robust study design (i.e., cRCT); external assessors for data collection at baseline and endline who were blinded to arm allocation of clusters; samples with similar characteristics recruited in both arms; and minimal loss-to-follow-up rate in both arms. A major strength of the intervention package is its better replicability and scalability, as it was integrated into the existing public health care and the messages on child development (both cognitive and physical) were contextually adapted in a way that required minimal additional resources.

Our study indicates that LMICs with reasonably functioning primary health care structures in place can adapt integrated ECD care interventions to address ECD delays. Our experience of successfully integrating ECD care into MCH at public-funded primary health care facilities is a significant step toward addressing the challenge of early development delays, mainly through staff skill enhancement and with almost no additional capital investment. Our experience suggests that integrated ECD care can be successfully sustained and scaled under routine health care circumstances. Our study implies that a countrywide scaling can possibly prevent global development delays in around one-quarter of children aged 12–24 months.

Our experience of successfully integrating ECD care into MCH at public-funded primary health care facilities is a significant step toward addressing the challenge of early development delays.

### Limitations

There are some limitations of the study. First, the health care providers and clients were not blinded to arm allocation of clusters, which could lead to bias and error. Second, the control arm was also strengthened for diagnosis and recordkeeping (same as the intervention arm) to a level higher than routine care to ensure standardized measurements across arms. This could have caused better outcomes in control as compared to actual routine care (without any intervention) due to better measurement and recordkeeping components. This would tend to reduce the effect size detected but not affect the results.

## CONCLUSION

The integrated ECD care package for public health facilities was found to be effective, leading to improvement in development delays and growth outcomes within the low-resource setting of Pakistan. The pragmatic study design shows that LHVs can successfully integrate ECD into their routine work. Therefore, the study indicates promising evidence for the scale-up and further integration of ECD interventions in the existing primary and secondary care in this South Asian context.

## Notes

**Peer Reviewed**

**First published online:** October 23, 2023.
